# Report of Medical Cases at the Hospital of the Sisters of Charity

**Published:** 1863-06

**Authors:** J. R. Lothrop


BUFFALO

### Medical and Surgical Journal

VOL. II. JUNE, 1863. NO. 11.

### ORIGINAL COMMUNICATIONS.

ART. I.—*Report of Medical Cases at the Hospital of the Sisters of Charity.* By J. R. Lothrop.

The following cases were under observation during an attendance of three months. This period is too short for the presentation of statistical information of much value in chronic cases, which in their results necessarily extend over long periods. In such cases the statistics of hospitals are frequently too favorable; the more so the shorter the time of observation. This is owing to the fact that in many instances persons afflicted with chronic diseases such as are from their nature, in the end, fatal, from want of means or disappointed in the amount of benefit received, leave the hospital; and hence are not entered on its mortuary record to swell its per centage of mortality. For long periods, however, the results of treatment in hospitals will not perhaps differ materially from the average in cases which are treated outside of the hospital. For, if in acute cases, by removal at a critical period of disease a fatal result is sometimes produced, on the other hand the improved hygienic conditions which the hospital affords, contribute largely towards a favorable result when removal has not been too long delayed—so that perhaps one is a fair offset to the other. It must be borne in mind in considering the ratio of mortality for a short period, that acute cases are more fatal at one season than another, and it may happen that for the period chosen, cases of unusual gravity fell under treatment. This is familiar to all in such diseases as scarlatina and dysentery, which are not only more fatal one year than another, but have this character at particular times in the year.

The exact results of treatment, in cases not fatal, cannot be stated except in a general way, without minute observation and report of individual cases. A general statement only can be made. Some remarks will be made upon classes of diseases after the statistical statement. One is prepared to expect recoveries or deaths in a certain proportion of such acute cases as have a termination one way or the other, in a comparatively short period. Without particular statement, recovery is to be presumed in typhoid fever, pneumonia, delirium tremens, and dysentery, if the fatal termination is not recorded. On the other hand, in phthisis, epilepsy, organic lesions of the heart, and cirrhosis, with the same reservation, the probability of improvement would be generally considered slight indeed. The cases which receive no benefit, which are in other words *not improved* by treatment, belong to the above and kindred diseases in their nature largely incurable. The numbers under the head of not improved will always be relatively large in hospitals, in which incurables are likely to accumulate.

With these observations as to their value and the inferences to be drawn from them, the following statistics and remarks are presented:

CASES RECEIVED FOR TREATMENT AND DEATHS.

| DISEASE. | MALE. | FEMALE. | TOTAL. | DIED. M. | DIED. F. | TOTAL. |
|---|---|---|---|---|---|---|
| Phthisis, | 11 | 3 | 14 | 1 | 1 | 2 |
| Typhoid Fever, | 10 | 1 | 11 | 3 | 1 | 4 |
| Intermittent " | 1 | 2 | 3 | | | |
| Remittent, " | 2 | 1 | 3 | | | |
| Scarlatina, | 4 | 1 | 5 | 3 | | 3 |
| Measles, | 1 | | 1 | | | |
| Delirium Tremens, | 11 | | 11 | 4 | | 4 |
| Pneumonia, | 1 | 1 | 2 | | | |
| Chronic Bronchitis, | 1 | | 1 | | | |
| Disease of Heart, | | 3 | 3 | | | |
| Gastric Disturbance, | | 3 | 3 | | | |
| Cirrhosis, | 2 | | 2 | 1 | | 1 |
| Jaundice, | | 1 | 1 | | | |
| Diarrhœa, | 6 | 5 | 11 | | 1 | 1 |
| Dysentery, | 2 | | 2 | 1 | | 1 |
| Rheumatism, | 3 | 3 | 6 | | | |
| Constipation, | 2 | 1 | 3 | | | |
| Neuralgia, | | 1 | 1 | | | |
| Menorrhagia, | | 2 | 2 | | | |
| Amenorrhœa, | | 3 | 3 | | | |
| Hysteria, | | 2 | 2 | | | |
| Anæmia, | | 1 | 1 | | | |
| Pelvic Hæmatocele, | | 1 | 1 | | | |
| Epilepsy, | | 1 | 1 | | | |
| Hydrocephalus Chronic, | 1 | | 1 | | | |
| Psoriasis, | 1 | | 1 | | | |
| Spinal Paralysis, | 1 | | 1 | | | |
| Syphilis, secondary, | 2 | | 2 | | | |
| Unknown, | 1 | | 1 | 1 | | 1 |
| | 63 | 36 | 99 | 14 | 3 | 17 |

The summary of results is as follows:

| | Males. | Females. | Total. | |
|---|---|---|---|---|
| Cases admitted, | 63 | 36 | 99 | |
| Died, | 14 | 3 | 17 | ratio nearly 1 in 6 |
| Recovered, | | | 44 | " " ½ |
| Improved, | | | 16 | " " 1 in 6½ |
| Not improved, | | | 22 | " " 1 in 4½ |

If we look at some of the most important diseases we find the ratio to be very nearly as follows:

| | | | |
|---|---|---|---|
| Phthisis, | Cases 14 | 2 deaths, | or 1 in 7 |
| Typhoid Fever, | " 11 | 4 " | or 1 in 3 |
| Scarlatina | " 5 | 3 " | or more than ½ |
| Delirium Tremens, | " 11 | 4 " | or 1 in 3 |
| Dysentery, | " 2 | 1 " | or 1 in 2 |
| Diarrhoea, | " 11 | 1 " | or 1 in 11 |

It will naturally occur that the period of observation was too brief for positive inferences in cases of phthisis, the ratio being by far too favorable. Again, the ratio in scarlet fever is far above the average; the cases, as will be seen, being of great severity—in fact mostly cases of scarlatinal dropsy. As a standard of comparison of results for a long period, the following statistics of St. George's Hospital, London, are given.

During six years the report of the medical wards of St. George's Hospital gives the ratio in

| | | | |
|---|---|---|---|
| Phthisis | whole number of cases 794 | per cent. of mortality 33, | or 1 in 3 |
| Typhoid Fever | " " " 911 | " " 11, | or 1 in 9 |
| Scarlatina, | " " " 79 | " " 12, | or 1 in 8 |
| Delir. Tremems, | " " " 93 | " " 18, | or 1 in 5½ |
| Dysentery, | " " " 21 | " " 42, | or 1 in 2 |
| Diarrhoea, | " " " 219 | " " 7, | or 1 in 14 |

To this may be added the writer's experience in another hospital, in which, during four years the number of cases under observation of the diseases mentioned above, is stated below. The list is made to include, in addition, cases of typhus or ship fever, which came under observation at the same time:

| | | | |
|---|---|---|---|
| Phthisis, | cases, 93 | deaths, 59 | ratio, more than ½ |
| Typhoid Fever, | " 84 | " 14 | " 1 in 6 |
| Typhus " | " 123 | " 19 | " nearly 1 in 6½ |
| Scarlatina, | " 13 | " 0 | |
| Delirium Tremens, | " 64 | " 1 | " 1 in 64 |
| Dysentery, | " 40 | " 16 | " nearly 1 in 2½ |
| Diarrhoea, | " 45 | " 5 | " 1 in 9 |

It is necessary to add that as the hospital was for the poor, few were discharged, unless well improved. There were fewer changes. This is evident in the ratio of mortality in phthisis. Typhus fever has about the same ratio as typhoid under equally favorable hygienic conditions. The fact that no death is set down to scarlatina is due probably to the occurrence of a few cases of light type, at different periods, among the children resident in the hospital. The light mortality in delirium tremens will be spoken of further on.

COMPARISON OF MORTALITY.

| | Sisters. | St. George. | Writer's cases. |
|---|---|---|---|
| Phthisis, | 1 in 7 | 1 in 3 | more than ½ |
| Typhoid Fever | 1 in 3 | 1 in 9 | 1 in 6 |
| Scarlatina, | more than ½ | 1 in 8 | |
| Delirium Tremens, | 1 in 3 | 1 in 5½ | 1 in 64 |
| Dysentery, | 1 in 2 | 1 in 2 | 1 in 2½ |
| Diarrhoea, | 1 in 11 | 1 in 14 | 1 in 9 |

*Phthisis.*—The occurrence of two deaths in fourteen cases gives a result far more favorable than the average. Eleven of the cases left the hospital after a stay of a longer or shorter time, and one only remained under treatment at the end of three months. This statement will explain the apparently small ratio of deaths. In only one case was the tubercular deposit in the lower part of the lung, the left lung; in the others it was at the apex of the lung, two in the right and the others in the left apex. No case of hæmoptysis.

*Typhoid Fever.*—Of the four fatal cases two were moribund when received. Two were complicated with purpura, and were both fatal. In these cases large blue spots resembling ecchymoses appeared on various parts of the body. There was, in addition, bleeding from all the mucous surfaces. Two were complicated with pneumonia. In all, diarrhœa occurred early. One died from perforation of intestine, attended in the last hours with vomiting of copious dark greenish fluid. The treatment was mainly supporting—nutritious diet, mostly milk and beef essence, and alcoholic stimulants, whiskey being the form selected usually. Opium was used to restrain diarrhœa, and also as an anodyne in cases in which nervous irritation was considerable. Quinine is highly recommended, and much given in this disease, directly to hasten convalescence. The belief is very general that it not only improves symptoms, but abbreviates the disease.—This belief does not rest on very certain grounds, and it seems clear from a summary of results as put forth by its advocates, that it is, at any rate, if of any value, only secondary. It may be of use to alleviate symptoms in certain forms of the disease, or in particular epidemics perhaps. The symptoms which may be benefited are headache, delirium, and somewhat, the agitation or twitching when they indicate disordered nervous action, rather than great prostration. In the latter state in which there is stupor, sliding down in bed, picking at imaginary objects and muttering, it is more likely to embarrass the stomach and aggravate the symptoms just spoken of. All effects claimed for it are those arising from large doses; in small doses no peculiar benefits are claimed for it. Large doses are certainly not free from a liability to redden the tongue, increase thirst, cause vomiting, and even to increase intestinal irritation, while they may aggravate certain spmptoms, such as stupor and tendency to coma, especially in adynamic forms of the disease.

For cutting short the disease in its early stages, or even producing a slightly earlier convalescence, it ought not to be given with any expectation that the end can be accomplished. In cases in which there is a blending of remittent and typhoid fever, it may be more successful. In malarious districts, diseases may be so influenced by miasma, as to call for the specific action of quinia, but many practitioners are prone to assume the necessity when no indications exist. Quinine is probably more valuable for its anti-miasmatic powers, than simply as a tonic, for which it is hardly as valuable as the bark itself. In the small doses in which it is often given, as a tonic, it is probably inferior to an equivalent amount of the bark. A small quantity given in the combination with its associated principles, which exists in the natural drug, is more efficacious as a mere tonic, than when used alone. In large doses it has an undoubted sedative action on the nervous system, as well as the pulse. Hence the benefit which is sometimes perceived, when the nervous symptoms are ataxic. But even in these cases, it should be employed with discrimination, and not as a mere matter of routine.

*Intermittent Fever.*—The single case was one of return of the paroxysms of intermittent after an interval of three weeks. The administration of quinine breaks up the regular succession of chills and fever, when it has accomplished so much its further use is not indicated. In many cases, as in this one, there is a return of the paroxysm in two or three weeks, when a second administration of quinine effectually averts the pyrexia. The quantity required is seldom less than 15 grains, in divided doses, a few hours apart, and often more. The time of giving is any time during the apyrexia, provided an interval of 8 or 10 hours is allowed between the last dose and the time of the expected attack. Many prefer to begin in the sweating stage, and there is probably no valid objection to it.

*Remittent Fever.*—Three mild cases were under treatment. In this as in intermittent, quinine has peculiar curative power, though less marked.—

It is to be given in the remission. But it will often occur that while the gastric irritability so often observed in the beginning continues, quinine produces no benefit, and often seems to increase the fever and gastric disturbance. A brisk cathartic, especially as there is usually constipation, of colocynth combined with blue pill, is an excellent preparative for quinia. This, of course, applies to the milder forms; malignant cases admit of but little preparation.

In this disease preparative treatment is more useful than in intermittent, but even in the latter, time enough is often afforded in the early part of the intermission, for the action of a cathartic before commencing with quinine, though this should be subordinate to interrupting the periodical succession of the paroxysms.

*Scarlatina.*—Of this disease five cases are reported and three deaths. Of the five, four should be classed under the head of sequelæ of scarlet fever. These four cases were of one family, of ages varying from 12 to 2 years. The youngest had the dropsy, which follows the primary disease but recovered. The other three died—one, the oldest, from exhaustive suppuration of the throat—the two others from dropsy and the accompanying suppression of urine.

The treatment adopted in the cases of dropsy was first, cathartic doses of calomel; secondly, tannin, and persulphate of iron successively. Calomel produced catharsis, but the other remedies were not always retained, vomiting being one of the symptoms present. The warm bath was also employed, and is in such cases always a remedy productive of benefit. No inferences as to the effects of the other remedies can be made. The case of recovery took place during the use of the iron. In cases of albuminuria connected with that change in the kidney known as Bright's disease, both remedies have been found useful in diminishing the amount of albumen in the urine; but the latter, viz: the iron in such cases, particularly when anæmia is marked often exerts a decided benefit, if not competent to effect a cure. Cases reported by Dr. White in this Journal, in which this remedy was employed, are of great interest. Scarlatinal dropsy is usually of short duration, and would often disappear without treatment. There is always an uncertainty as to the effect of remedies in diseases which tend to recovery in a very short time. It may be found that this form of iron is a useful medicine in this affection.

*Delirium Tremens.*—The fatal cases were not all uncomplicated delirium tremens. One was complicated with epilepsy, or epileptiform convulsions—

one with severe diarrhœa, one was moribund when received, so that the diagnosis was that stated by his attendants. One only could be said to have died from nervous exhaustion after prolonged violent delirium, i. e. of uncomplicated delirium tremens. But in this case there may have been organic disease with it, and at any rate there was a constitution shaken by excessive intemperance and previous attacks of delirium tremens. The violence of the delirium, without marked tremor, might give rise to a suspicion of cerebral disease, aggravated by stimulants.

One can hardly speak of delirium tremens as a disease, meaning thereby to indicate a definite order of phenomena always to be met with, any more than he can direct a course of treatment always to be adopted. Under the name are included various forms of mental disorder which require different methods of treatment. The true form of the disease, that in which there is tremor, sweating and delirium, in which there is seldom violence, but restlessness and fear, in which the illusions are tinged with gloom and apprehension, is confounded with that in which there is more violence, often almost maniacal excitement. The last is more likely to be the direct exciting effect of alcohol on the brain, and is usually accompanied by signs of recent and excessive use, the patient having a strong smell of alcohol. The first results from deprivation of the usual amount of stimulus from some cause, which may be inability to procure it, as in prisoners, locked up after a prolonged debauch; or restriction imposed by friends; or, and this is the most common cause, the rejection of the liquor by the stomach, so that the customary amount is not absorbed. The brain equally falls into disorder if the amount of alcohol in contact is too large or too small, but the symptoms are different, and so must be the management. If there is evidence of recent and excessive drinking, a strong smell of alcohol about the patient, and violent delirium without marked tremor, sweating or alarm at all about him, no course can be more proper and rational than to await the elimination of the poison, aiding by a cathartic, or even emptying the stomach if a large quantity has been recently taken. For here the mental disorder is the direct toxemic effect of alcohol. In many cases this suffices. But it is not to be supposed a resort to stimulants may not be afterwards necessary to prevent the patient's falling into the true delirium, especially if he has been drinking for some time, or has used alcohol habitually. This is to be left to the good sense of the physician; but primarily the expectant course is the proper one. Setting aside now such cases, in which the error is often committed of giving opium and alcohol, the true delirium requires to be noticed.

In this the symptoms are of the ataxic character, arising from the deprivation of an habitual stimulus to the nervous system, hence the muscular movements, the impressions upon the senses, and the mental operations are all disordered, causing trembling, illusions, and delirium. The most rational course in treatment in such cases is, to restore the accustomed stimulus in moderate quantities. Some other interference by drugs may occasionally relieve certain symptoms, as for instance vomiting, but the essential curative agent is one which affords to the nervous system the stimulation it needs. Various substances may have this effect—alcohol, ether, chloroform, and in certain doses opium. The erroneous idea which is at the foundation of treatment in these cases, is that sleep, which is the natural termination of the disease, should be procured as early and as directly as possible, i. e. by measures tending to bring about this end immediately. Hence the strong tendency to interfere by narcotics. Opium may cause a sleep from which the patient will not waken. It is true that large doses often will have no perceptible influence, but in these instances the cause is probably rather in deficient absorbing power of the stomach, than a want of susceptibility to the action of the drug. The object of treatment should be to favor early recovery, not merely to procure sleep as the only object, for it may be the sleep of death. Our measures should have this ultimate aim to correct disordered nervous action by the necessary amount of stimulation, and to accomplish this opium in proper doses, not large, is as efficient perhaps as the alcoholic stimulants. When its effect passes beyond its prime action, namely, nervous stimulation, its benefit is questionable. Given in this way it relieves the intensity of certain symptoms, quiets the alarm and restlessness, though its immediate influence is not to cause sleep. In the natural course of things there is a tendency to termination in sleep at the end of a few days, usually three days, or seventy-two hours, if the vigilance has been constant; if broken by snatches of sleep the period may be prolonged. The method of treatment above mentioned favors this tendency, it cannot be said to do more, and in fact it will in practice be found difficult to do more; it being by no means easy to shorten the duration of an attack.

In the cases reported the treatment adopted consisted of stimulants, whiskey being the form mostly employed, though Hoffman's Anodyne in some cases, in dram doses, at intervals of a few hours, was sometimes either substituted for it, or combined with it; and opium in doses of one or two grains, repeated twice or three times at intervals at night. Cathartics if used at all were always employed at the outset, but then according to the indications. There is often irritability of stomach and constipation which a cathartic overcomes, and after it food is better retained and digested.— Ten or fifteen grains of comp. ext. of colocynth and five grains of blue pill combined with a grain or two of hyosciamus or conium was as satisfactory as any cathartic. Podophyllin was tried, but from some cause did not act well. The diet consisted largely of concentrated animal essences and milk. The patient was urged to take as freely as possible of beef essence, especially if not much food had been taken for several days.— Restraint was avoided as far as was consistent with the peace of the ward. The amount of stimulation to be employed must depend upon the nature of the case, asthenic cases requiring most, while young men in tolerable vigor require least. A patient in whom the disease appears for the first time needs less than one who has suffered from repeated attacks.

It is difficult to estimate the benefit of remedies and to determine how far the result in a favorable case is due to nature, but if we resort to treatment it seems most rational to look upon the disease, true delirium tremens, as a depression of the nervous system from deprivation of an habitual stimulus. When the plan of leaving the attack to pursue its course to its natural termination has been adopted, results have been attained which compare very favorably with those observed when stimulants or drugs have been used. But the plan can only be adopted in public institutions. In private a physician is in a measure forced to employ remedies. The friends are anxious, and could not be kept quiet during several days and nights of unceasing vigilance and delirium on the part of the patient. The expectant plan will not answer for them.

It will be observed that in the cases reported by the writer the mortality wss 1 in 64. It should be stated in explanation that the death was set down to delirium tremens only when the case was uncomplicated. There may have been among the 64 cases other fatal cases, in which acute diseases, or organic lesion had the principal agency in causing death, though delirium was present; but on this point the writer has no distinct remembrance, as the notes were made a number of years since. So small a proportion of fatal cases is not contrary to the experience of others. Dr. Gerhard states that in the Blockley Hospital, the mortality in successive years was 1 in 26, 1 in 34, and 1 in 160. The above result of 1 in 64

may be almost entirely claimed as the result of the expectant plan of treatment. The course pursued and rarely departed from, was to give food as freely as it could be taken, and keep the patient in a well lighted room, with the least restraint possible. As to medication nothing except a cathartic was usually employed, and stimulation by alcohol or opium very seldom resorted to, i. e. there was little active medication. This was the usual plan, but it is not to be understood that there was never a variation. It cannot be claimed that an expectant plan can be always followed, for the course of the disease may call for interference, but it can be with good results, in many cases which are indiscriminately subjected to a routine treatment of alcohol and opium. The results in cases in which either opium or stimulants were the principal remedies, are given in the following statements of Dr. Ware and Dr. Gerhard. The former states, that of 15 cases treated mainly by opium 6 died, while of 45 cases treated mainly without opium and without active remedies, only 2 were fatal. Dr. Gerhard advocates the use of alcoholic stimulants, and abandonment of opium as a principal remedy. His statement is, that while under the full use of opium the mortality was about 1 in 10; after its disuse it fell to the figures quoted above, or from ten to less than one per cent.

*Diseases of the Heart.*—Three cases are recorded, all occurring in females, between the ages of 17 and 21, and all of rheumatic origin; confirmatory of the observation that metastasis of rheumatism to the heart is more frequent in young persons. In two cases the lesion was insufficiency of the mitral valves in the third aortic obstruction, in each with more or less hypertrophy.

*Gastric Disturbance.*—Under this indefinite heading are included three cases of vomiting and irritability of stomach, the result of the rather free use of alcoholic stimulants, occurring in females.

*Cirrhosis.*—One fatal case, and one discharged from the hospital, of course unimproved. In the fatal case dropsy and jaundice were associated. In this case the effect of the retained bile elements to cause passive hæmorrhage was well marked. Bleeding took place from all the mucous surfaces, and wherever the skin was broken. This was arrested very effectually by the persulphate of iron in doses of 10 drops a few times daily.

*Diarrhœa.*—Of the cases mentioned one was complicated with delirium tremens, and contributed largely to the fatal issue. One was chiefly interesting as having been contracted in the "swamps of the Chickahomany,"

and though not treated as were our soldiers in that noted campaign, on the expectant plan, it received but little benefit from any treatment.

*Rheumatism.*—Three acute and three chronic cases were received. In two the smaller joints were affected, the variety being rheumatic gout.—Two were complicated with phthisis. In one, the case of a young woman, there was cardiac metastasis, resulting in mitral lesion. In one case Tr. of aconite was given, and with much relief to the pain. In two, scruple doses of nitrate of potash three times daily, combined with twenty drops of the Tr. of digitalis, were attended with benefit, i. e. with sweating, allaying of pain and decrease of redness and swelling about the affected joints. This last combination even in large doses is often productive of decided benefit. It is in fact alkaline treatment, but probably no better if as good, as the alkaline carbonates or acetates or tartrate of soda and potash, which have proved highly serviceable.

*Constipation.*—One case, arose probably from cancerous constriction of the descending colon, as the dilated and distended intestine could be felt through the abdominal walls. The stricture is usually in or near the sigmoid flexure. In these cases as in cases of ileus, the intestine becomes paralyzed by distention with the gradually accumulating contents, so that the most active cathartics fail to produce an expulsion of the accumulated matter, though they cause pain and griping in the intestines above. In this case there was vomiting, and the patient stated that there had been constipation for six weeks. Repeated enemas succeeded in removing the accumulation, but the most active cathartics failed. The patient was a woman of fifty, of most cachectic appearance. She did not remain long in the hospital.

For habitual constipation various trials have proved the value of podophyllin in small doses of one-half to one grain, in some cases combined with ext. of nux vomica $\frac{1}{2}$ grain and ext. of conium $\frac{1}{2}$ to 1 grain, once, twice or thrice daily, as the case demands.

*Pelvic Hæmatocele.*—This case was reported in a past number of this Journal. Perfect recovery took place. The pathology of the disease is yet in uncertainty. It is a question whether the peritoneum forms part of the sac, or whether the extravasation is beneath it. In all fatal cases which have been examined the peritoneum has been found to form a part of the sac, the extravasation being then above it. From this it might be inferred that it was so in all cases. But it is more probable that those in which the extravasation is above the peritoneum are alone fatal, while those in which it is beneath, recover.

*Epilepsy.*—The single case of epilepsy was in a young girl 17 years of age. There was an interval of one or two weeks between the epileptic seizures, then several convulsions succeeded each other at short intervals, during which the tongue was bitten, and the face became spotted by ecchymoses, especially about the outer angle of the eyes. The fits were succeeded by prolonged sleep. During the interval the health was apparently good, though there was an evident loss of memory. Epilepsy coming on about the time of puberty is generally considered most favorable, i. e. if not associated with some evident centric or eccentric cause. While under treatment the case received no benefit, though subjected to various medication. Most practitioners look upon a cure by medical means as almost hopeless, though there has been considerable testimony in favor of several medicines. An entire abstinence from animal diet, i. e. flesh, is a measure almost always advisable, unless the state of health renders it obviously improper. The testimony in favor of stramonium as a remedy in this disease, renders its claims worthy of attention, though they are not, we must confess, as positive as could be desired. One would hardly look for *curative* action, but when used for any length of time, and in sufficient doses to slightly affect vision, by dilating the pupil, it will in many cases prevent the frequent recurrence of convulsions. The cases suited to it are those in which the cause is hereditary, or those in which if no hereditary predisposition can be made out it cannot be traced to injury of any part of the nervous system, to any disease which by reflex action could act as a cause of nervous irritation, or even to great or sudden mental excitement; cases in fact, belonging to the so-called idiopathic epilepsy.

*Chronic Hydrocephalus.*—This case was benefited in so marked a manner by the iodide of potash, that there seemed to be good reason for believing that, as has been claimed, it is of use in such cases. The case had been under treatment three months, and probably mercury had been given; but improvement followed soon after the iodide was given, and complete recovery took place after two months' use.

---

The report of the death of Dr. George Suckley, Medical Director of the Eleventh Army Corps, is incorrect; Dr. S. remained with the wounded, and is a prisoner.

